# Supplementary material for: Three-Dimensional Shear Wave Elastography Using a 2D Row Column Addressing (RCA) Array
Source: BME Front. 2022 Jul 4;2022:9879632. doi: 10.34133/2022/9879632 (PMC10521701; doi:10.34133/2022/9879632)
Supplement: Supplementary Materials — Figure. S1: shear wave speed maps of the (a) stiffer spherical lesion and (b) background of the elasticity phantom using the GE LOGIC E10 system and a 1D linear array. The measurement was conducted in ARF-based 2D SWE mode. Figure. S2: mechanical index of the RCA array as a function of the input voltage. The measurements were performed using a needle hydrophone in the water tank, and the RCA transmitted a focused beam using 64 elements (F−number=2) or 128 elements (F−number=1), and the focal depth is 25 mm. Figure. S3: reconstructed volumetric images (slice view) of the phantom using four different transmission schemes (compounding plane wave imaging, synthetic aperture imaging (SA), focused beam imaging (FB), and wide beam imaging (WB)). (a) Compounding plane wave imaging with RC scheme with an angular pitch of 1°, angular aperture of 15°, 31 compounding angles, and 400 Hz volume rate. (b) Compounding plane wave imaging with CR scheme with an angular pitch of 1°, angular aperture of 15°, 31 compounding angles, and 400 Hz volume rate. (c) Compounding plane wave imaging with RC+CR scheme with an angular pitch of 1°, angular aperture of 15°, 62 compounding angles, and 200 Hz volume rate. (d) SA imaging with 128 transmissions and 100 Hz volume rate. one element was used for each transmission. (e) SA imaging with 128 transmissions and 100 Hz volume rate, virtual source (a subaperture of 12 elements) was used for each transmission. (f) SA imaging with 64 transmissions and 200 Hz volume rate, virtual source (a subaperture of 64 elements) was used for each transmission. (g) FB imaging with 64 transmissions and 200 Hz volume rate, 32 elements were used for each focused beam with a F-number of 4. (h) FB imaging with 128 transmissions and 100 Hz volume rate, 32 elements were used for each focused beam with a F-number of 4. (i) WB imaging with 30 transmissions and 400 Hz volume rate, 50 elements were used for each subregion compounding. (j) WB imaging with 60 transmissions and [file 9879632.f1.zip › BMEF_RCA_3D_SWE_Supplementary.pdf]

SUPPLEMENTARY MATERIALS

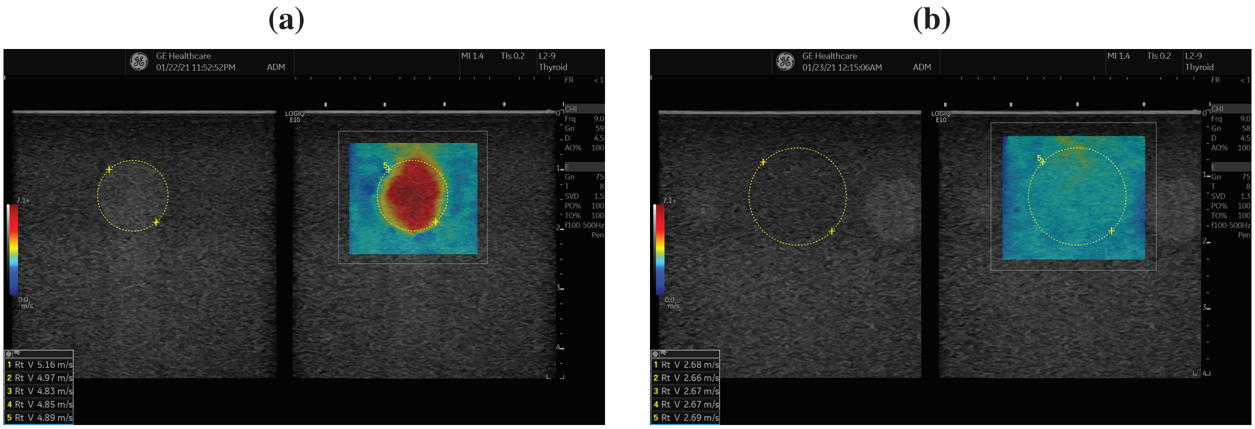

Figure. S1 Shear wave speed maps of the (a) stiffer spherical lesion and (b) background of the elasticity phantom using the GE LOGIC E10 system and a 1D linear array. The measurement was conducted in ARF-based 2D SWE mode.

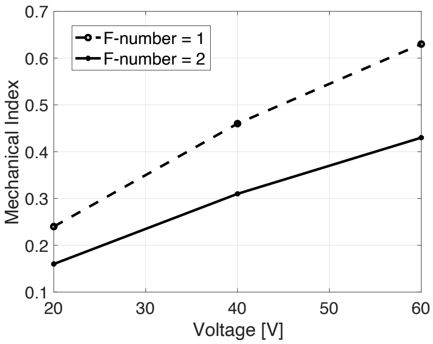

Figure. S2 Mechanical index of the RCA array as a function of the input voltage. The measurements were performed using a needle hydrophone in the water tank, and the RCA transmitted a focused beam using 64 elements (F-number = 2) or 128 elements (F-number = 1), and the focal depth is 25 mm.

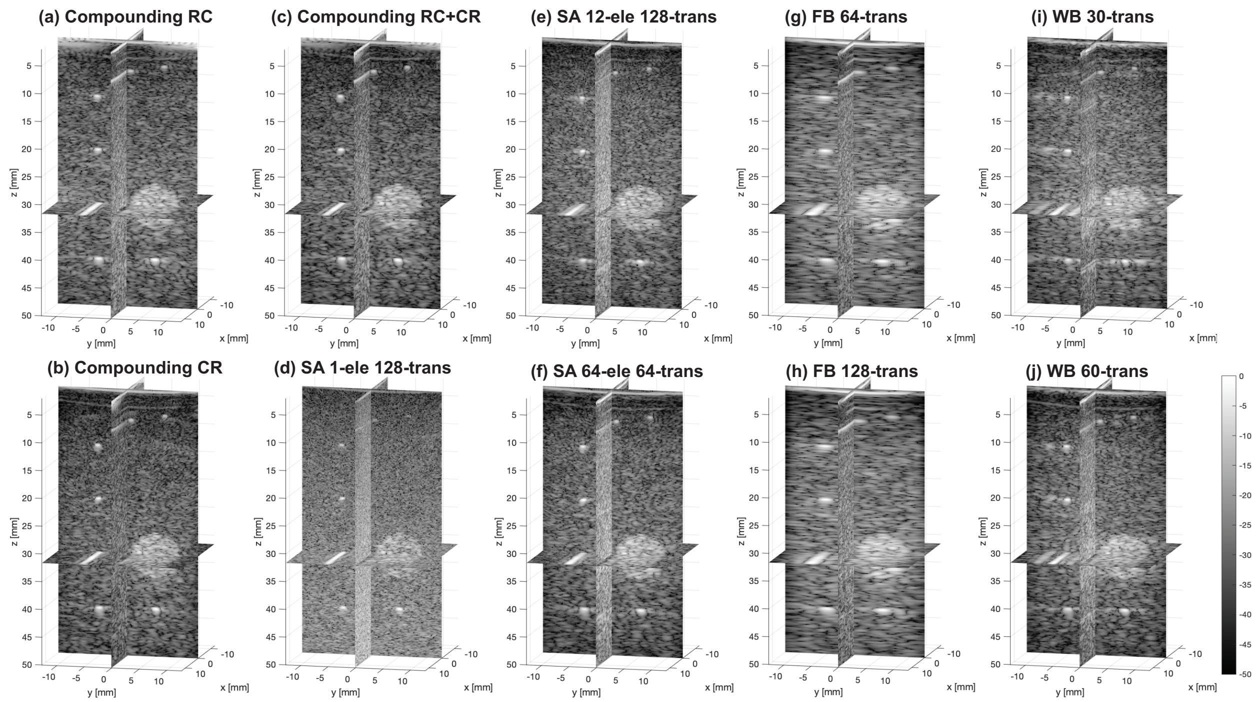

Figure. S3 Reconstructed volumetric images (slice view) of the phantom using four different transmission schemes (compounding plane wave imaging, synthetic aperture imaging (SA), focused beam imaging (FB), and wide beam imaging (WB)). (a) Compounding plane wave imaging with RC scheme with an angular pitch of $1°$, angular aperture of $15°$, 31 compounding angles, and 400 Hz volume rate. (b) Compounding plane wave imaging with CR scheme with an angular pitch of $1°$, angular aperture of $15°$, 31 compounding angles, and 400 Hz volume rate. (c) Compounding plane wave imaging with RC+CR scheme with an angular pitch of $1°$, angular aperture of $15°$, 62 compounding angles, and 200 Hz volume rate. (d) SA imaging with 128 transmissions and 100 Hz volume rate, one element was used for each transmission. (e) SA imaging with 128 transmissions and 100 Hz volume rate, virtual source (a sub-aperture of 12 elements) was used for each transmission. (f) SA imaging with 64 transmissions and 200 Hz volume rate, virtual source (a sub-aperture of 64 elements) was used for each transmission. (g) FB imaging with 64 transmissions and 200 Hz volume rate, 32 elements were used for each focused beam with a F-number of 4. (h) FB imaging with 128 transmissions and 100 Hz volume rate, 32 elements were used for each focused beam with a F-number of 4. (i) WB imaging with 30 transmissions and 400 Hz volume rate, 50 elements were used for each sub-region compounding. (j) WB imaging with 60 transmissions and 200 Hz volume rate, 50 elements were used for each sub-region compounding.
